# The complete chloroplast genome of *Gymnospermium kiangnanense* (Berberidaceae): an endangered species endemic to Eastern China

**DOI:** 10.1080/23802359.2018.1483760

**Published:** 2018-07-03

**Authors:** Zhaoping Yang, Zhengkang Peng, Hongwei Zhang, Joongku Lee, Xuelian Liu, Chengxin Fu

**Affiliations:** a Key Laboratory of Conservation Biology for Endangered Wildlife of the Ministry of Education, Laboratory of Systematic & Evolutionary Botany and Biodiversity, College of Life Sciences, Zhejiang University, Hangzhou, China;; b College of Life Sciences, Tarim University, Alaer, China;; c Administration of Zhejiang Qingliangfeng National Natural Reserve, Hangzhou, China;; d Department of Environment and Forest Resources, Chungnam National University, Daejeon, South Korea;; e College of Life Science, Tonghua Normal University, Tonghua, China

**Keywords:** Gymnospermium kiangnanense, Berberidaceae, chloroplast genome, phylogenetic tree

## Abstract

*Gymnospermium kiangnanense* is an endangered species in the family Berberidaceae. In this study, we assembled the chloroplast (cp) genome of *G. kiangnanense* and reconstructed the phylogenetic tree of Berberidaceae based on the complete cp genome. The cp genome of *G. kiangnanense* is 160,136 bp in length, comprising a pair of inverted repeat (IR) regions (26,424 bp) separated by a large single-copy (LSC) region (87,580 bp) and a small single-copy (SSC) region (19,708bp). The genome encodes 111 unique genes, consisting of 77 different protein-coding genes, 30 transfer RNA, and 4 ribosomal RNA genes, with 18 duplicated genes in the inverted repeats. Phylogenetic analysis indicates that *G. kiangnanense* is sister to *G. microrrhynchum*, and the *Gymnospermium* clade is closely related with *Nandina*.

*Gymnospermium* Spach (Berberidaceae) is a small genus containing about 12 species (Barina et al. 2017). Most of the species are native to temperate Europe and Asia (north of N37°), while *G. kiangnanense* (P.L. Chiu) Loconte is the only one that distributed far south in the subtropical area (between N29° and N31°). *G. kiangnanense* is endemic to Anhui and Zhejiang Provinces of China, with very few known sites (Chiu 1980). Due to the high medicinal and ornamental values, *G. kiangnanense* has been overexploited for decades. Thus, Zhejiang Provincial Government (2012) designated it as a key protected plant. To further understand its genetic background, we assembled the first complete chloroplast genome of *G. kiangnanense* and the sequence was registered in the GenBank with the accession number MH298010.

Total genomic DNA was isolated from silica-dried leaves of a transplanted *G. kiangnanense* plant in Qingliangfeng Botanical Garden, China (118°54′05.14″E, 30°06′34.23″N), using DNA Plantzol Reagent (Invitrogen, Carlsbad, CA) according to the manufacturer’s protocol. Voucher specimen (*Pan Li LP161264-1*) was deposited at the Herbarium of Zhejiang University (HZU). Then the high molecular weight DNA was sheared (yielding ≤800 bp fragments) and the quality of fragmentation was checked on an Agilent Bioanalyzer 2100 (Agilent Technologies, Palo Alto, CA). The short-insert (500 bp) paired-end libraries preparation and sequencing performed on Illumina HiSeq 2500 with read length of 125 bp (Beijing Genomics Institute – BGI, Shenzhen, China). The raw data (approximately 1.37 Gb) were filtered by quality with Phred score <30 and all remaining sequences were assembled into contigs using CLC Genomic Workbenck 10.1.1 (QIAGEN Bioinformatics, Aarhus, Denmark). All the contigs were aligned to the reference chloroplast genome (*G. microrrhynchum*, NC_030061.1) using BLAST (NCBI BLAST v2.2.31) search. The representative chloroplast contigs were ordered and oriented according to the reference chloroplast genome. Finally, clean reads re-mapped to the draft genome and yielded the complete chloroplast genome sequences. The phylogenetic tree was reconstructed based on the complete cp genome sequences of 15 Berberidaceae species and one outgroup, using maximum likelihood (ML) method. ML analysis was implemented in RAxML-HPC v8.2.10 (Stamatakis 2014) on the CIPRES cluster (Miller et al. 2010).

The complete chloroplast genome of *G. kiangnanense* is 160,136 bp in length and shares the common feature of comprising two copies of IR (26,424 bp each) that divide the genome into two single-copy regions (LSC 87,579 bp; SSC 19,709 bp). The whole GC content of the total length, LSC, SSC, and IR regions is 37.8%, 36.1%, 31.0%, and 43.1%, respectively, which is similar to that of the chloroplast genomes reported for the other Berberidaceae species (Zhang et al. 2016; Meng et al. 2017). Within the cp genome of *G. kiangnanense*, there are 111 unique genes, including 77 protein coding genes, 30 tRNA genes, and four rRNA genes, with 18 genes duplicated in the IR regions. The phylogenetic tree showed that *G. kiangnanense* was sister to *G. microrrhynchum*, and the *Gymnospermium* clade was closely related with *Nandina domestica* (Figure 1).

**Figure 1.**
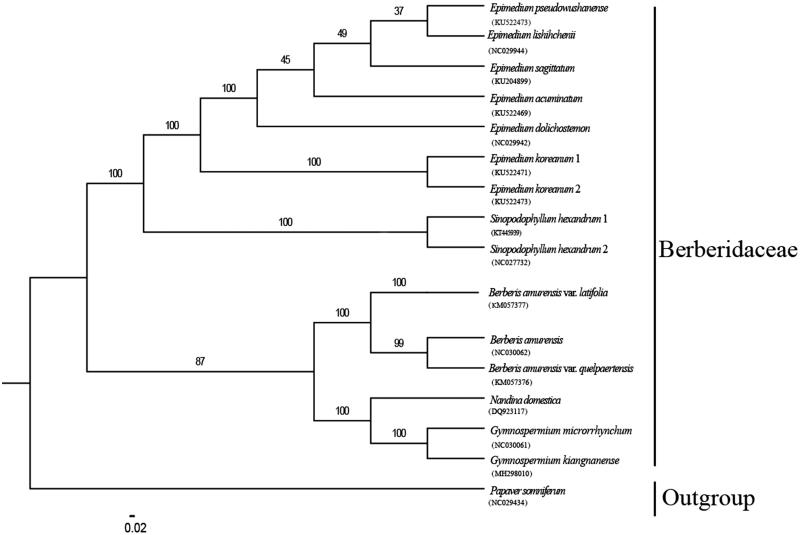
Phylogenetic tree reconstruction based on complete chloroplast genome sequences of 15 Berberidaceae taxa and one outgroup, using maximum likelihood (ML) method.

In conclusion, this is the first report of the complete chloroplast genome of *G. kiangnanense*. It will provide useful genetic resources for future conservation of this endangered species, and also shed light on the evolution of the family Berberidaceae.
